# Immunomodulation Potential of Probiotics: A Novel Strategy for Improving Livestock Health, Immunity, and Productivity

**DOI:** 10.3390/microorganisms10020388

**Published:** 2022-02-07

**Authors:** A. K. M. Humayun Kober, Muhammad Shahid Riaz Rajoka, Hafiza Mahreen Mehwish, Julio Villena, Haruki Kitazawa

**Affiliations:** 1 Laboratory of Animal Food Function, Graduate School of Agricultural Science, Tohoku University, Sendai 980-8572, Japan; shahidrajoka@yahoo.com (M.S.R.R.); mahreen.mehwish@yahoo.com (H.M.M.); 2 Livestock Immunology Unit, International Education and Research Centre for Food and Agricultural Immunology, Graduate School of Agricultural Science, Tohoku University, Sendai 980-8572, Japan; 3 Department of Dairy and Poultry Science, Chittagong Veterinary and Animal Sciences University, Khulshi, Chittagong 4225, Bangladesh; 4 Laboratory of Immunobiotechnology, Reference Centre for Lactobacilli (CERELA-CONICET), Tucuman 4000, Argentina; jcvillena@cerela.org.ar

**Keywords:** antimicrobial, livestock, healthy growth strategy, probiotics, immunoregulatory effects of probiotics

## Abstract

Over the past decade, the use of probiotics as feed supplements in animal production has increased considerably due to the ban on antibiotic growth promoters in livestock. This review provides an overview of the current situation, limitation, and prospects for probiotic formulations applied to livestock. Recently, the use of probiotics in livestock has been suggested to significantly improve their health, immunity, growth performance, nutritional digestibility, and intestinal microbial balance. Furthermore, it was reported that the use of probiotics in animals was helpful in equilibrating their beneficial microbial population and microbial turnover via stimulating the host immune response through specific secretions and competitive exclusion of potentially pathogenic bacteria in the digestive tract. Recently, there has been great interest in the understanding of probiotics targeted diet and its ability to compete with harmful microbes and acquire their niches. Therefore, the present review explores the most commonly used probiotic formulations in livestock feed and their effect on animal health. In summary, this article provides an in-depth knowledge about the formulation of probiotics as a step toward a better alternative to antibiotic healthy growth strategies.

## 1. Introduction

**Figure 1:**
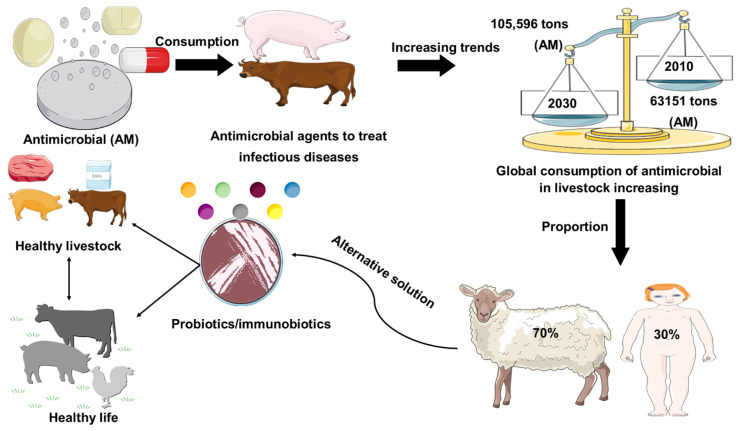
Role of probiotics in livestock healthy growth strategy. Global consumption of AM (AM) in livestock production was estimated in 2010 and is projected to rise by 67%, by 2030. Global increase (67%) in AM consumption is due to the growing number of animals raised for meat and milk production. Probiotics used as a safer alternative to conventional antibiotic drug therapy.

Antimicrobial resistance represents a global health problem that contributes to tens of thousands of deaths per year. Furthermore, the global demand for meat and dairy consumption is increasing at a rapid and unprecedented rate [1]. To fulfill this demand, many countries are shifting to intensive livestock production systems that use antimicrobial (AM) drugs to keep animals healthy and increase their development and productivity [2,3]. For example, Van Boeckel et al. (2015) found that between 2010 and 2030, the global consumption of AM agent for livestock industry increased by 67%, while on the other hand, the increase in AM agent consumption in the BRICS countries (Brazil, Russia, India, China, South Africa) will be 67%. Furthermore, Denmark was the foremost nation to report authorized antimicrobial agent manufacturing/sales data in 1996, under the name of Danish Integrated Additive Manufacturing Resistance Monitoring and Research Program (DANMAP). In 2011, the European Medicines Agency Surveillance of Veterinary Consumption group (ESVAC) published the first report on veterinary AM sales in eight countries (Czech Republic, Denmark, Finland, France, Netherlands, Norway, Sweden, UK) since 2005. The latest 2017 report provides an overview of AM sales across all EU countries. Furthermore, North American countries and Canada began collecting sales data for AM resistance monitoring in 2008 for the Canadian Comprehensive Program (CIPARS), which reports AM resistance and AM use. In Asia, Japan was the first country to launch the Japan Veterinary AM Monitoring System (JVARM) to report AM agent use [4]. In addition, current global trends in the use of AM agents in livestock animal feeds were represented in Figure 1. Therefore, the establishment of AM-free feeding system by using probiotics has been required for secure and healthy livestock production. The most commonly used probiotics in livestock are the strains of lactic acid bacteria (LAB) and *Bifidobacterium* [5]. In addition, gastrointestinal tract (GI) infections in livestock are considered a major global problem, with a negative economic impact on livestock farmers [6]. In this regard, the likelihood of using feed supplements to attain a healthier animal, welfare, and yield by manipulating the gut microbiota has received considerable attention over the past 30 years. Antibiotics have been applied widely to prevent and treat GI infection in livestock; however, the random uses of antibiotics in livestock are responsible for the development of antibiotic resistance, which has a long-lasting effect on the human body, as well as the destruction of gut microflora [7,8,9]. Probiotics might be used as a potential alternative therapy to treat gastrointestinal tract disorders and to enhance the endogenous immune function of the host (Figure 1).

Numerous probiotics might be used to improve the performance of ruminant and pig (Table 1, Table 2 and Table 3) animals. Numerous studies have demonstrated that probiotics can exert an AM effect against pathogens and improve animal health, as well as productivity [10,11]. Earlier, our group established a porcine intestinal epithelial (PIE) cell line and demonstrated that PIE cells are a useful in vitro tool for the selection of immunomodulatory LAB (immunobiotic LAB). Furthermore, our group has demonstrated that the in vitro and in vivo immunobiotic LAB is a good alternative to improve resistance against GI pathogens in the porcine host. Additionally, our laboratory has shown that the probiotic *Lactobacillus* with immunoregulatory functions can beneficially modulate the immune response in the gut through controlling the functions of PIE cells (Table 2 and Table 3) [10,11,12,13,14,15]. This contrasts with previous studies that recommend the modulation of gut microbiota and piglet immunity via appropriate probiotic strains, which will lead to better growth performance. Therefore, it is necessary to establish a non-toxic feeding system and a food safety system to ensure the safe and healthy production of animal husbandry. A recent study suggested that the probiotic-supplemented diet significantly improved the health status, growth performance, and intestinal morphology in pigs [16]. Similarly, it was suggested that the multi-species probiotic diet has excellent potential to endorse the growth performance and healthy status of pigs via modulation of gut microbiota [17].

Therefore, this review brings forward a summary of recent scientific literature, as well as its implications in terms of animal health and productivity of the main species of farm animals, such as pigs, cattle, goats, and sheep. This review also explores the mechanism of actions of immunomodulation by probiotic LAB in intestinal epithelial cells (IECs) in in vitro animal model.

## 2. Global Trends of Antimicrobial Uses in Livestock

The growing global demand for animal protein consumption is putting increasing pressure on bacteria to develop AM resistance. It was recently reported that the average annual global consumption per kilogram of animal AM agents utilized was within the range of 45 mg and 172 mg in cattle and swine, respectively [63]. Additionally, it has been estimated that the global AM consumption will increase by 67% (from 63,151 tons to 105,596 tons) in between 2010 and 2030, respectively (Figure 1). In between 2010 and 2030, the rapidly increasing trends for consumption of livestock have put pressure on shifting the production practices in developing nations, where extensive agribusiness systems will be replaced by extensive agricultural procedures [4]. For BRICS countries, the AM consumption trends will increase by 99%, which is higher than the projected citizen income growth in these nations. In India, for example, the consumption proportion of AM agents (30 kg per square kilometer) is likely to upsurge to 312% by 2030. Strangely, almost 70% of AM agents, considered medically important for human health by the FDA, were used in livestock in US, ultimately threatening human health and animal welfare [64]. Hence, many countries, such as the EU and Japan, have banned the use of AM agents as growth promoters. In addition, other countries, including China and the US, are planning to ban the addition of antibiotics to animal feed, and research will focus on growth promoters that do not affect human health [65]. A potential alternative solution to these problems is the addition of naturally derived dietary additives, such as probiotics, that have enhancing effects on livestock health and productivity (Table 1, Table 2 and Table 3). Recent research with probiotic LAB in livestock suggested that the LAB might be used as an alternative strategy to antibiotic growth promoters [11,18,32,59,60,61,62].

## 3. Application of Probiotics in In Vivo Studies for Pig Production

The microorganisms most commonly used as probiotics in pigs are presented in Table 1 and Table 2. The genus *Lactobacillus* might be considered one of the most prominent components of the host gut. Furthermore, to date, no such report on safety precautions linked to the use of *Lactobacillus* in swine had been compiled. In growing-finishing pigs, the supplementation of probiotics has shown beneficial effects on the intake of feed alongside animal average weight. Furthermore, the addition of probiotics significantly reduced the activity of blood complement, while no alterations have been noted in antibody levels, macrophages, and leukocytes activities [18,19]. On the other hand, the probiotic treatment might be helpful in enhancing the growth performance, quality, and productivity in livestock [11,20,21] (Table 3).

Oral supplementation of probiotics to neonates alters early mucosa-associated colonization patterns in preterm piglets and, hence, reducing the mucosal atrophy and gut dysfunction, including diarrhea, which is one of the most serious gastrointestinal disorders disturbing preterm piglet neonates [22,23]. Furthermore, piglets are very susceptible to colonization of the gut by pathogenic microorganisms that cause growth retardation and diarrhea, starting from birth to post-weaning. Therefore, probiotics are suggested throughout this time period, and many studies have shown the effectiveness of such products [11]. It was suggested that the probiotic (*L. reuteri*, *B. subtillis*, and *B. Licheniformis*) supplementation of pigs after weaning enhances the performance of animals [24]. It was concluded that the probiotic supplementation was helpful in promoting a healthy intestine by improving the digestibility of the nutrient, reducing the fecal *Salmonella*, as well as *E. coli* contents, improving serum IgG level and probably also resistance to local infection. It was also reported that dietary supplementation with 0.1% (1.5 × 10^9^ CFU/g) probiotics (*B. coagulans*, *B. licheniformis*, *B. subtilis* and *C. butyricum*) can improve growth performance, nutrient digestibility, blood profiles, and it can modulate the concentrations of *Lactobacillus* and *E. coli* and decrease fecal noxious gas emission in weaning pigs [66].

Kantas et al. (2015) reported that *B. toyonensis* improved health, as well as growth performance, and could protect against enteric pathogens in post-weaning piglets [25]. Multi-strain probiotics at 0.1% concentration might be used as an alternative to a growth-promoting strategy [26]. The probiotic LAB demonstrated excellent ability in decreasing the pathogen microbe intestinal colonization, as well as exciting local immune response and enhancing intestinal architecture [27,67] in piglets. On the other hand, the origin of probiotic strains, the dose of probiotics in different husbandry enhances, piglet age, and feed system might present contrasting outcomes with the same probiotic strains [68]. The results of Masumizu et al. (2019) suggest that *L. salivarius* FFIG131 and *L. salivarius* FFIG71 might be used as immunobiotic candidates for the development of new immunological feed in the future, which in turn enhance pay to enlightening immune health status in the porcine host [29]. *B. subtilis PB6* feeding in weaning pigs increased feed efficiency and boosted immunity, along with reducing fecal ammonia and diarrhea [30].

The in vivo study by Islam et al. (2021) revealed that immunobiotic feeding resulted in significant positive health benefits when using rakkyo pickled juice fermented with *L. plantarum* N14 in pigs, thus increasing feed intake, efficiency, and carcass quality [31]. In addition, another study by Suda et al. (2021) suggested that the immunobiotic *L. delbrueckii* subsp. *Delbrueckii* TUA4408L okara feed significantly improved growth performance and meat quality in piglets [32]. Tian et al. (2021) investigated the influence of diet supplemented with *L. reuteri 1* (*LR1*) or antibiotics (olaquindox and aureomycin) on the longissimus thoracis (LT) and concluded that the probiotic might enhance health condition and carcass quality of treated pigs as compared with the control group [33]. Diet supplementation with probiotics *B. subtilis PB6* enhanced growth performance and immunity, alongside lowering ammonia emissions and diarrhea incidence [30]. Furthermore, the *L. plantarum* supplemented diet significantly enhanced growth performance, increased *Lactobacillus* fecal contents, and decreased *E. coli* counts in weaning pigs [34,69].

## 4. Application of Probiotics in In Vivo Studies for Ruminant Production

Probiotics for mature ruminant animals have primarily been chosen for targeting the rumen compartment, which would be the primary site of feed digestion [70]. A wide variety of strictly anaerobic bacteria, ciliate protozoa, anaerobic fungi, and archaea constitute the rumen microbial ecosystem, which is responsible for the breakdown and fermentation of 70–75% of food components [71,72]. Live yeast (*Saccharomyces*) formulations are by far the most commonly marketed products for ruminants [73,74]. Live yeasts have been proven to boost performance in dairy ruminants by improving their immunity [75].

Daily live yeast supplementation has also been shown to increase growth metrics (such as average daily weight gain, final weight, and food intake) in beef cattle [39,76]. Most of these effects have been linked to increasing overall culturable ruminal microbial population concentration, which leads to an enhanced activity of cellulolytic microorganism growth and increased fiber digestibility. Numerous previous studies indicated that probiotics in ruminants increased their performance [77,78], although some studies [79] found little or no changes. Furthermore, a recent study suggested that the symbiotic supplementation of a yeast-derived prebiotic and a *B. subtilis* significantly improved the health conditions and overall productivity during feedlot receiving period [80]. Additionally, it was demonstrated that the supplementation of probiotic yeast products improved the inflammatory response of cattle on these diets. In conclusion, overall benefits of *Saccharomyces*-based products on animal performance may be linked to improved cattle health and increased nutrient digestibility [81].

The probiotic was given to calves throughout their first month of life, and it enhanced their gut microbiota and growth performance, as well as some biometric parameters [41]. Hence, a high quantity of compound probiotics is suggested to progress rumen development and health status of Holstein calves [82]. It has been reported that probiotics containing *Bacillus* spores and nucleotides had no synergistic impact on calves’ development, welfare, or fecal bacteria; however, nucleotide supplementation reduces *Lactobacillus* feces levels [83].

Lambs that received probiotics in a post-weaning nourishment seemed to exhibit a better performance in terms of feed conversion ratio, growth performance, and nutrient digestibility [44]. Probiotics administered orally to dairy cows exhibited a systemic effect on gene expression, including genes involved in immunity and homeostasis [45]. Zhang et al. (2016) aimed to develop a diet enriched with *L. plantarum* and *B. subtilis* on Holstein calves, and the developed diet with *L. plantarum* improved growth performance, nutrient digestibility, and relieved weaning stress in calves [43]. In the case of buffalo calves, the supplementation with *L. acidophilus* increased body weight gain and feed efficiency [46,84]. The study suggests that the fermented milk containing LAB can be beneficial for young calves because of its positive impacts on health and growth [46,84]. *Ruminococcus flavefaciens* supplementation in feed increased production performance in sheep [48]. It has also been stated by Izadi et al. (2020) that the probiotic *B. coagulans* can be used as an improving factor to increase the quality of milk and of dairy foodstuffs [79,85].

## 5. Application of Probiotics Using Cell Lines as Livestock Animal Model (In Vitro Study)

**Figure 2:**
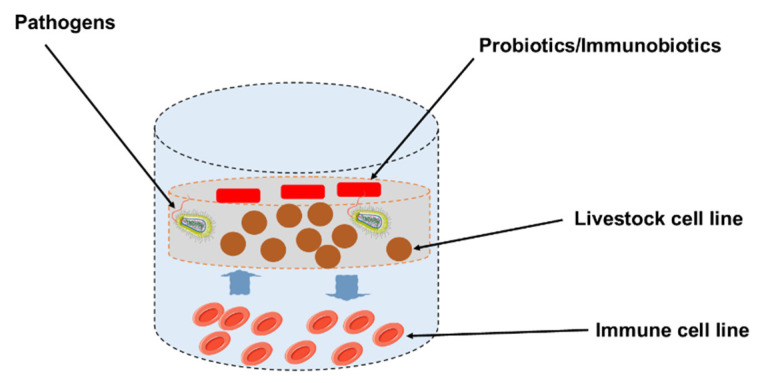
The in vitro cellular research model for the assessment of immunomodulatory function of probiotics/immunobiotic in livestock.

The in vivo studies indicate that probiotics have been successfully used to improve growth performance, nutrient utilization, intestinal microbiota, and gut health of the main species of farm animals, such as pigs, cattle, goats and sheep (Table 1 and Table 2). Some functional feeds that contain probiotics are thought to improve intestinal immunity via inspiration of epithelial cells, as well as immunocompetent cells, through pattern recognition receptor and induction of cytokine in the GIT [86,87]. However, in the field of feed immunology, due to the unavailability of an adequate intestinal immunoassay system for farm animals, much about the underlying mechanisms of intestinal immunity in cattle remains unknown. As a result, developing a probiotics/immunobiotic evaluation system for probiotic supplementation of functional food to a farm animal model is critical. In these circumstances, our group developed porcine and bovine intestinal epitheliocyte (PIE and BIE) cell lines for the evaluation of probiotics/immunobiotics and immunogenicity using anti-inflammatory responses in PIE cell monolayers and a co-culture system with porcine Peyer’s patch immune cells as a Peyer’s patch culture model (Figure 2) [12,54,88,89,90].

Our work demonstrated that the intestinal epitheliocytes (PIE, BIE) are useful in vitro model systems for the assessment of relations between pathogens and porcine/bovine intestinal epithelial cells (IECs), for the selection of probiotic/immunobiotic microorganisms, and for the evaluation of underlying immunomodulatory mechanisms by probiotic LAB in IECs. Currently, our study and a few other in vitro studies focused on describing the “health-improving” activities of probiotics in farm animals, along with effects of immune-health promoting factor (Table 3).

Treatment with *L. acidophilus* (LA) before rotavirus infection boosted PRV replication and IL-6 response to PRV infection, indicating that LA had an adjuvant effect. Following rotavirus infection, LGG therapy reduced the IL-6 response, indicating LGG’s anti-inflammatory properties in an IPEC-J2 cell line [50]. It was reported that *L. casei* MEP221106 significantly regulate the antiviral immune response in PIE cells via TLR3-mediated immune response [90].

Fujie et al. (2011) found that in a PIE cell line, *B. breve* MCC-117 has the ability to effectively control the inflammatory response produced by enterotoxigenic *E. coli* (ETEC). They also found that MCC-117 has excellent immunoregulatory activity, which was linked to strain ability to alter PIE and the interaction of immunological cells, resulting in the stimulation of regulatory T cells and prevention of ETEC-induced intestinal inflammation [12]. On the other hand, another study indicated that *L. jensenii* TL2937 significantly reduced pro-inflammatory cytokines and chemokine expression caused by ETEC, leading to prevention of inflammatory intestinal disorders [54]. Subsequently, Tomosada et al. (2013) showed that *B. longum* BB536 and *B. breve* M-16V strains reduced the expression of intereleukin-8, interleukin-6, and MCP-1 in PIE cells treated with heat-killed ETEC [10].

Similarly, Takanashi et al. (2013) showed that *L. casei* OLL2768 reduced inflammation in PIE cells by reducing the production of pro-inflammatory cytokines [52]. Furthermore, Abedi et al. (2013) demonstrated that *L. delbrueckii* exhibited excellent ability to inhibit *E. coli* infection in the gut by using Caco-2 cells [51]. Furthermore, *L. jensenii* TL2937 was reported to be able to stimulate the production of immunoregulatory factors, such as TGF- in EICs, and functionally modulate IECs to improve infection resistance and minimize non-protective inflammation [11]. Our study suggests that feed supplemented with *B.* *thermophilum* stimulates immune cells to exert immunoregulation, which indicates that this feed is likely to contribute to enhancing the health of piglets without using AM feed materials [55].

Kang et al. (2015) indicated that *L. ruminis* SPM0211, *B. Longum* SPM1205, and *B. longum* 1206 are proficient in preventing the in vitro and in vivo rotavirus replication. Additionally, it was suggested that the antiviral effects of probiotics are to be expected owing to their modulation of the immune response via regulation of type I IFNs [57]. Another study reported the ability of LAB to beneficially modulate the inflammatory response in PIE cells after being challenged with pathogenic bacteria ETEC and virus (poly (I:C)) and to modulate gut immunity in the porcine host [29]. Another recent study demonstrated that the *L. delbruecki* TUA4408L attenuate ETEC-induced inflammatory response in PIE via TLR-2 and ETEC-induced inflammatory cytokines were downregulated when PIE cells were pre-stimulated with TUA4408L [91]. A recent study by Kobayashi et al. (2017) proved that the *B. infantis* MCC12 or *B. breve* MCC1274 have the ability to lower RV titers in BIE cells and differentially control the innate immune response. Furthermore, it was indicated that the bacterial strains enhanced the antiviral factor production, such as IFN-β in RV-infected BIE cells. In addition, recently we reported that *L. rhamnosus* CRL1505 and *L. plantarum* CRL1506 are immunobiotic strains with the ability to enhance the fortification against viral intestinal infections, as demonstrated in PIE [15].

The PIE cell stimulation with poly (I:C) enhanced the production of *IFN-α* and *IFN-β*, chemokines, adhesion molecules, cytokines, and prostaglandin biosynthesis genes. CRL1505 and CRL1506 modulate the innate antiviral immune response in PIE cells and protect against viral infection and inflammatory damage in vivo [92]. Another recent study by Kanmani et al. (2018) demonstrated that *L. delbrueckii* OLL1073R-1 modulate the innate antiviral immune response in porcine intestinal epithelial cells [59]. A recent study by Iida et al. (2019) demonstrated that paraimmunobiotic *Bifidobacteria* (*B. longum* BB536 and *B. breve* M-16V) can be used as a substitute to enhance intestinal infection resistance or as therapeutic gears for decreasing the inflammation [60]. Mizuno et al. (2020) demonstrated that *L. plantarum* CRL1506 significantly enhanced the intestinal innate antiviral immune response [61]. Śliżewska et al. (2021) demonstrated that new *Lactobacillus* strains might be helpful in preventing intestinal infections by reducing the colonization of pathogenic bacteria [62]. As a result, the use of probiotic *Lactobacillus* strains may improve the safety and quality of animal-derived meat and food products. Therefore, previous studies suggest that the use of immunobiotics/probiotics has good potential for immunomodulation to prevent and improve different health disorders.

### Limitation for the Use of Probiotics in In Vitro and In Vivo Research Model

It was shown that the in vitro studies have a variety of limitations that must be considered. Results obtained with different IECs, for example, must be taken with caution because not all cell lines have the same properties. It is also worth noting that culture circumstances can affect how some molecular traits are expressed. The molecular explanation of probiotic action in vivo will aid in the identification of authentic probiotics and in the selection of the most appropriate ones for disease prevention and/or treatment. Nevertheless, further studies are also required ① to determine whether the probiotics used in animal nutrition enter the human food chain and how they affect human health. ② Animal womb is in an aseptic state, but after birth, young animals are suddenly exposed to bacteria and virus. To prevent infection from pathogenic bacteria and viruses, young livestock develops immunogenic potential by acquiring not only immunoglobulin and cytokine from colostrum but also indigenous bacteria from the mother’s vagina and milk. Among them, if useful immunobiotics for raising animals without AM agents can be found, they will be safe for animals as well as humans. Therefore, more investigation will be required in order to find *Lactobacillus* in the form of immunobiotics, pursue the possibility of using them as AM substitutes, and try to construct immunobiotics library to establish the translocation route from mother to child, which will represent the translocation route of indigenous bacteria from mother to child. Further studies are also required ③ to elucidate the mechanisms of action of probiotic LAB strains—in particular those related to the immunoregulating ability of LAB strains through DCs activation via pattern recognition receptors (co-culture experiments with probiotics, DCs, and IECs as well as in 3D models); ④ to search for probiotics that can be used as drug alternatives in the prevention or treatment of various infectious diseases using in vitro and in vivo models; ⑤ to search for new techniques, such as genome editing and AI/IoT system, for the development of a healthy growth system with immunobiotics.

## 6. Application of Probiotics in Livestock Production

In recent decades, some studies were conducted to illustrate the new scope in the field of probiotics and to discover the potential probiotic microbes. According to Sun et al., (2021) multi-species probiotics consisting of *L. acidophilus, L. casei*, *B. thermophilum*, and *E. faecium* were successfully used to reduce the diarrhea caused by enterotoxigenic *E. coli* (ETEC) F18^+^ in newly weaned pig [93]. In addition, multi-species probiotics were helpful in enhancing growth performance through a reduction in intestinal inflammation, oxidative stress, and morphological damages. Sobrino et al. (2021) attempted to study AM substitutes in pig production. They used *Ligilactobacillus salivarius* strain retrieved from sow’s milk and fed it to pregnant sows and piglets. The results suggested that there was a notable reduction in the presence of antibiotic-resistant *Lactobacillus*, which became apparent in the treatment group [94]. In recent studies, it was suggested that *Prevotella* exerted positive consequences in pig production by enhancing growth performance and immune response [95,96,97,98]. The *Lactobacillus, Escherichia, Shigella*, and *Bacteroides* dominate the small intestine microbiota, while on the other hand, the *Prevotella* dominates the large intestinal microbiota during the newborn stage. Furthermore, the *Prevotella* dominates the pig’s small and large intestines after weaning [99]. Additionally, it was reported that the non-diarrheic piglets were found to have a considerably higher abundance of intestinal *Prevotella* than diarrheic piglets. *Prevotellacea* UCG-003 was the key bacterium in the non-diarrheic microbiota of piglets, according to co-correlation network analysis [98]. Ngo et al. (2021) used a new probiotic (*B. amyloliquefaciens* H57) in high concentrate feed pellets that reduces volatile fatty acid production and prevents flavor in pellet feed. That facilitates higher feed intake in ruminant animals [28]. In recent studies on anaerobic fungi, it was demonstrated that it contributes essentially to ruminal fiber utilization by degrading plant cell walls in two ways, i.e., enzymatically and mechanically [100,101]. Remarkably, ongoing exploration showed the affinity of fungal CAZymes for stubborn fiber, which might clarify the specific use of anaerobic fungi when lower quality forages were fed to ruminants. Therefore, this can also be used as a potential probiotic in ruminant nutrition [102]. Studies on the utilization of *B. subtilis* as a spore-shaping probiotic bacterium in livestock nutrition have shown no unsafe impacts and have exhibited the viability of its utilization as a probiotic, mostly because of its demonstrated AM, mitigating cell reinforcement and exhibiting enzymatic, and immunomodulatory action [103]. A study by Cai et al. (2021) enumerated that *S. cerevisiae* and *C. butyricum* and their blend enhanced rumen conditions by expanding the pH and diminishing oxidation and upgraded rumen maturation capacities by expanding absorbability of supplements and further developing VFA production; from that point on, further enhancements in production growth of heat-stressed goats were observed [104]. The *Debaryomyces hansenii* is also gaining attraction as a new potential probiotic for both terrestrial and aquatic animals. The oral delivery of *D. Hansenii* has been linked to probiotic features, such as immunostimulatory effects, gut microbiota regulation, increased cell proliferation, differentiation, and improved digestive function. Its bioactive molecules have been identified and linked to its immunomodulatory effect, including cell wall components and polyamines [105]. Therefore, there are many potential probiotic microbes that are still to be discovered, which might play an evolutionary role in livestock production.

## 7. Modes of Action of Livestock Probiotics

**Figure 3:**
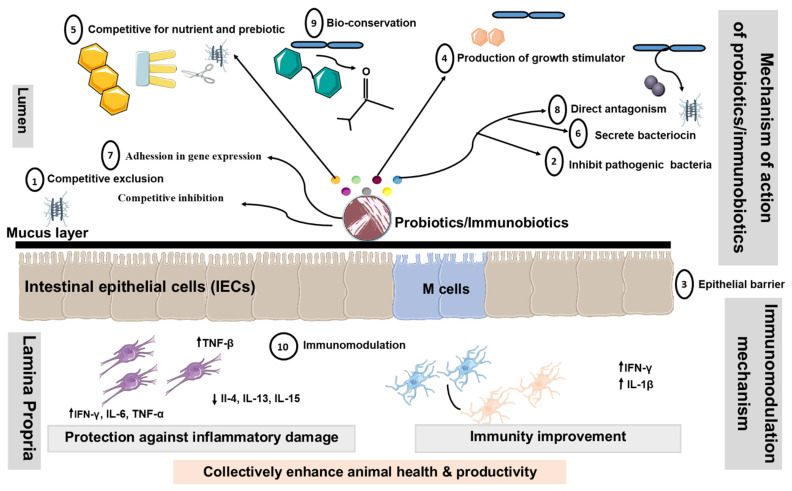
Proposed modes of action of livestock probiotics. Schematic diagram illustrating potential mechanisms, whereby oral administration of probiotics might promote beneficial effects by changing the composition of intestinal microbiota, altering intestinal barrier function, bile salts, and production of Th1 cytokines. Additionally, probiotics containing LAB may down-regulate the expression of pro-inflammatory cytokines and chemokines. Decrease in the translocation of bacteria may occur as a result of the ability of probiotics to tighten the mucosal barrier. Probiotics disallow colonization by pathogenic bacteria through competition for nutrients, immune system up-regulation, and production of antitoxins. These mechanisms include ① Competitive exclusion for binding sites, ② Adhesion to the GIT,③ Enhancement of the epithelial barrier, ④ Increase in digestion and absorption of nutrients, ⑤ Competing with pathogenic bacteria for nutrients in the gut, ⑥ Production of AM substances, ⑦ Alteration in gene expression in pathogenic microorganisms, ⑧ Bacterial antagonism, ⑨ Bioconversion and ⑩ Immunomodulation. Abbreviations: ↑, increased; ↓, decreased; Th1, Type 1 T helper; Th2, Type 2 T helper; IEC, intestinal epithelial cells; DC: dendritic cell.

There are numerous proposed modes of action of livestock probiotics [106,107,108,109,110,111,112,113,114]. However, the major mechanisms of action proposed for probiotics are considered in the following segments (summarized in Figure 3).

① *Modification of the microbial population of the GIT*: Probiotics might boost the population of beneficial microbes, such as *Lactobacillus* and *Bifidobacterium*, which subsequently restrict the growth of harmful bacteria by creating inhibitory chemicals and by competing for binding sites [115,116]. ② *Adhesion to the GIT wall to prevent colonization by pathogenic microorganisms*: The majority of enteric pathogens might colonize the intestinal epithelium and cause disease as a result [117]. As a result, *Lactobacillus* can adhere to the gut epithelium and compete with pathogens for adhesion receptors, such as glycoconjugates [118]. The *Lactobacillus* and *Bifidobacterium* have hydrophobic surface layer proteins that assist the bacteria non-specifically by adhering to the animal cell surface [119]. ③ *Enhancement of the Epithelial Barrier:* The experimental studies in model animal have shown that probiotics *P. acidilactici* improve intestinal barrier function by reducing the permeability of the intestinal epithelium translocation of enterotoxigenic *E. coli* to mesenteric lymph nodes in post-weaning piglets as compared to the control group after ETEC challenge [120]. Our current findings suggest that the *L. jensenii* TL2937 reduce the intracellular Ca^2+^ flux in DSS-challenged PIE cells, increasing the tightness of the tight junction [121].

④ *Increase in digestion and absorption of nutrients*: In this case, the spore-forming bacteria enhance the production of extracellular enzymes, which facilitate nutrient digestion [122,123]. ⑤ *Competing with pathogenic bacteria for nutrients in the gut*: Probiotic bacteria might compete with pathogenic bacteria for nutrients and absorption sites by rapidly utilizing energy sources, potentially shortening the log phase of bacterial development [116]. ⑥ *Production of antimicrobial substances*: Several probiotic bacteria, particularly those that produce lactic and acetic acids, have the ability to suppress harmful microorganisms [124,125]. ⑦ *Alteration in gene expression in pathogenic microorganisms*: Probiotics might influence pathogenic bacteria’s quorum sensing, hence altering their pathogenicity. Fermentation products from *L. acidophilus* La-5 significantly suppressed the extracellular production of a chemical signal (autoinducer-2) by human enterohaemorrhagic *E. coli* serotype O157:H7, leading to inhibition of the virulent gene (LEE—locus of enterocyte effacement) expression in vitro [126]. ⑧ *Bacterial antagonism:* Probiotic microorganisms, once established in the gut, may produce organic acids, hydrogen peroxide, lactoferrin, and bacteriocin, which may exhibit either bactericidal or bacteriostatic properties [127].

⑨ *Bactericidal activity*/*Bioconversion*: *Lactobacillus* convert lactose to lactic acid, lowering the pH to a point where pathogenic bacteria cannot survive. Furthermore, living yeasts compete with lactic acid-producing bacteria to digest sugars obtained from starch breakdown, thereby stabilizing rumen pH and minimizing the danger of acidosis [128,129,130]. ⑩ *Immunomodulation*: Our study has shown that probiotic LAB with immunoregulatory functions can beneficially modulate the immune response in the gut by modulating the functions of PIE cells [12,54,56]. In addition, probiotic LAB have proven to be capable of acting as immune modulators by enhancing macrophage activity [54], increasing local antibody levels, inducing the production of anti-inflammation cytokines (interleukin (IL)- 10, interferon (IFN)-γ, β, IL-1β, TGF-β), reducing IL-4, IL-6, IL-8, MCP-1, and activating killer cells [11,32,54].

Immunomodulation properties appear to be strain dependent, which means that dissimilar probiotics might have parallel mechanisms of action, whereas a single strain may have multiple mechanisms of action. Quite a lot of probiotic strains, for example, have comparable impact on the microbial community of gastrointestinal tract, although the mechanisms of action of certain probiotics are mostly unknown. The exact mode of action of probiotics is not well understood in the majority of studies on their impact on performance. Therefore, the mechanisms must be explored on a case-by-case basis because closely interrelated probiotics appear to have diverse ways of action. Probiotic effects are a result of the interaction between the host and the probiotic microorganism. As a result, more research into the host–microbe interaction could shed light on the probiotic mode of action. Rapid improvements in molecular techniques and genome sequencing for microbial ecology research will substantially aid our understanding of probiotic mechanisms of action.

## 8. Conclusions

In the present review, we provided an overview of the effects of probiotics, including NGP on livestock in terms of nutrition, health, productivity, and the mechanisms of action of probiotics. Additional knowledge on the in vitro system of livestock animal model for the study of the mechanisms of immunomodulation by probiotic LAB in IECs is also illustrated. Several livestock probiotics have been found effective in improving animal weight gain, feed conversion, digestibility of nutrients, IgG, immune status, intestinal microflora and gut health (increased Lactobacilli with decreased *E. coli* counts), intestinal morphology, milk yield and quality, meat production and carcass quality, and reduction of the risk of pathogen colonization, stress, and diarrhea in both pig and ruminant livestock industries. Probiotics can be used as drug alternatives in growth promoters and in the prevention or treatment of various infectious diseases. Finally, in this review we also suggest that immunobiotics LAB can modulate immune responses in intestinal epithelial and immune cells from livestock, suggesting many potential probiotics could be discovered by new techniques, such as genome editing and AI/IoT system for contributions to promote healthy livestock without using AM feed materials, which ultimately will lead to drug-independent healthy and productive livestock, as well as food safety system for food animals.

## Figures and Tables

**Table 1:** Summary of current pig trials (in vivo) measuring the effects of probiotics on health and productivity.

Genus	Species/Strains	Age Group	Probiotic Effects in Respect to					Ref.
			Weight Gain/Feed Intake	Feed Efficiency	Health	Immunity	Others	
*Bacillus/Clostridium*	*B. subtilis* and *C. butyricum*	Growing-finishing pigs (GFP)	↑	↑	↑	-	↑ Meat qua	[18]
*Lactobacillus/Enterococcus*	*L. plantarum* ATCC 4336, *L. fermentum* DSM 20016 and *E. faecium* ATCC 19434	Weaned piglets (WP)	↑	↑	-	-	-	[19]
*Bacillus/Saccharomyces*	*B. subtilis* and *S. boulardi*	GFP	↑			-	↓ *E. coli*	[20]
*Lactobacillus*	*L.plantarum* ZJ316	GFP	↑	-	↑	↑	↑ Meat qua	[21]
*Bifidobacterium*	*B. longum* (AH1206)	Neonatal piglets	↔	↔	↑	↑	↑ Mucosal immune	[22]
*Lactobacillus*	*L. fermentum*	Suckling/nursery piglets	↑	↑	↑	↑	↓ Diarrhea	[23]
*Lactobacillus*	*L. reuteri*, *B. subtillis* and *B. licheniformis*	WP	↑	-	↑	↑	↓ Salmonella and *E. coli*	[24]
*Lactobacillus*	*L.jensenii* TL2937 (LjTL2937)	GFP	↑	↑	↑	↑	↑ Meat qua	[11]
*Bacillus*	*B. toyonensis*	WP	↑	↑	↑	-	↓ Enteric pathogens	[25]
*Bacillus/Clostridium*	*B. coagulans*, *B.licheniformis*, *B. subtilis* and *C. butyricum*	WP	↑	↓	↑	↑	↓ Fecal noxious gas emission	[26]
*Bifidobacterium*	*B. longum*	WP	↑	↑	↑	↑	↓ Intestinal colonization by pathogens	[27]
*Lactobacillus/Bifidobacterium*	*L.* spp., *B.* spp.	GFP	↑	↑	↑	-	↓ Post-weaning mortality	[28]
*Lactobacillus*	*L. salivarius FFIG71 L. salivarius FFIG131*	GFP	-	-	↑	↑	-	[29]
*Bacillus*	*B. subtilis PB6*	WP	↑	↔	↑	↑	↓ Fecal ammonia and diarrhea	[30]
*Lactiplantibacillus*	*L. plantarum N14*	GFP	↑	↑	-	↑	↑ Meat qua	[31]
*Lactobacillus*	*delbrueckii* subsp. *Delbrueckii TUA4408L*	WP	↑	↑	↑	↑	↑ Meat qua	[32]
*Lactobacillus*	*L. reuteri 1 (LR1)*	GFP	↑	↑	-	-	↑ Meat qua	[33]
*Lactobacillus*	*L. plantarum BG0001*	WP	↑	↑	-	-	↓ Fecal *E. coli*	[34]

**Abbreviations**: ↑, increased; ↓, decreased; ↔, no significant difference between groups; -, not studied; qua, quality.

**Table 2:** Summary of current ruminant trials (in vivo) measuring the effects of probiotics on health and production.

Genus	Species/Strains	Source	Probiotic Effects in Respect to					Ref.
			Weight Gain/Feed Intake	Feed Efficiency	Health	Immunity	Others	
-	Multi-species probiotic	Cattle	↑	↔	↑		-	[35]
*Enterococcus*	*E. faecium M74*	Calf	↑	-	↑	↑	-	[36]
*Bacillus/Saccharomyces*	*B. cereus S. boulardii*	Sheep				↑		[37]
*Kluyveromyces/Saccharomyces*	*K. marximanus NRRL3234*, *S. cerevisiae NCDC42*, *S. uvarum ATCC9080*	Sheep	↑	↑	-	-	-	[38]
*-*	Multi-species probiotic	Calf	↔	↔	↔	-	-	[35]
*-*	Multi-species probiotic	Cattle	↑	↔	-	-	-	[39]
*Saccharomyces*	*S. cerevisiae* or combination of *S. cerevisiae* and *L. sporogenes*	Sheep	↔	-	-	-	-	[40]
*-*	Multi-species probiotic LAB	Calves	↑		↑	-	↓ Weaning time	[39]
*Lactobacillus*	*L.animalis* SB310, *L.paracasei* subsp. *Paracasei* SB137 and *B.coagulans* SB117	Calves	↑	↑	↑	-	Improved gut microbiota	[41]
*Faecalibacterium*	*F. prausnitzii*	Calves	↑	↑	-	↑	-	[42]
*Lactobacillus*	*L. plantarum*	Calves	↑	↑	↑	↑	↓ Weaning stress	[43]
*Pediococcus*	*P*. *pentosaceus*	Sheep	↑	↑	↑	-	↑ Digestibility	[44]
*Lactobacillus*	*L. acidophilus*, *S. cerevisiae*, *E. faecium*, *A. oryza*, and *B. subtilis*	Cattle	-	-	↑	↑	-	[45]
*Lactobacillus*	*L. acidophilus*	Buffalo calves	↑	↑	-	-	↓ Fecal coliform	[46]
*Lactobacillus*	*L. murinus CRL1695, L. mucosae CRL1696, L. johnsonii CRL1693*, *L. salivarius CRL1702*	Calves	↑	↑	-	-	↓ Diarrhea and calf mortality	[47]
*Ruminococcus*	*R. flavefaciens*	Sheep	↑	↑	-	-	-	[48]
*Lactobacillus*	*L. sporogenes*	Calves	↑	↑	-	-	-	[49]

**Abbreviations**: ↑, increased; ↓, decreased; ↔, no significant difference between groups; -, not assessed.

**Table 3:** Summary of current trials in cell line as livestock animal model (in vitro) measuring the immunoregulatory effects of probiotics.

Genus	Species/Strains	Source	Expt. Mode	Time	Probiotic Effects in Respect to				Ref.
					Anti-Inflammation	Pro-Inflammation	Immune-Health	Others	
*Lactobacillus*	*L. acidophilus* (LA) or *L.* *rhamnosus* GG (LGG)	Dairy products	IPEC-J2	24 h	↓	↑	↑	↓ RVs titers	[50]
*Lactobacillus*	*L. casei* MEP221106	Korean food	PIE	48 h	↓	↑	↑	↑ AV immune	[51]
*Bifidobacterium*	*B. breve* MCC-117	Human gut	PIE	48 h	↓	↑	↑	↓ Intestinal inflammation	[12]
*Lactobacillus*	*L. casei* OLL2768	Dairy products	BIE	48 h	↓	↑	↑	↑ APP	[52]
*Lactobacillus*	*L. delbrueckii*	Yogurt	Caco-2	Overnight	-	-	↑	↓ *E. coli* infection	[53]
*Bifidobacterium*	*B. longum* BB536 and *B. breve* M-16V	Infant	PIE	48 h	↑	↓	↑	↑ APP	[10]
*Lactobacillus*	*L. jensenii* TL2937	Human feces	PIE	48 h	↑	↓	↑	↑ APP	[11,54]
*Bifidobacterium*	*B. thermophilum* and *B. infantis*/*B. brevis*	Feed	PIE	48 h	↓	↑	↑	-	[14,55]
*Lactobacillus*	*L. rhamnosus* CRL1506	Goat milk	PIE	48 h	↓	↑	↑	↑ AV	[56]
*Lactobacillus/Bifidobacterium*	*L. ruminis* SPM0211, *B. longum* SPM1205 and SPM1206	Human	Caco-2		↓	↑	↑	↑ AV immune	[57]
*Lactobacillus*	*L. salivarius* FFIG53	Pig intestine	PIE	48 h	↑	↓	↑	↑ APP	[29]
*Lactobacillus*	*L. plantarum* N14 and *L. delbrueckii* TUA4408L	Fermented pickle	PIE	48 h	↑	↓	↑	-	[58]
*Bifidobacterium*	*B.infantis* MCC12 or *B.* *breve* MCC1274	-	BIE	48 h	↓	↑	↑	↓ RVs titers	[15]
*Lactobacillus*	*L. delbrueckii* OLL1073R-1	Yogurt	PIE		↑	↓	↑	↑ AV	[59]
*Bifidobacterium*	*B. longum* BB536 and *B. breve* M-16V	Human feces	PIE	120 h	↑	↓	↑	-	[60]
*Lactobacillus*	*L. plantarum* CRL1506	Goat milk	PIE	72 h	↑	↓	↑	-	[61]
*Lactobacillus*	*Lactobacillus* spp.	Manure	Caco-2 cells	7–10 D	-	-	-	↓ Infections	[62]

**Abbreviations**: ↑, increased; ↓, decreased; -, not assessed/provided; APP, anti-pathogenic potential; AV, antiviral; RVs, rotavirus.

## Data Availability

Not applicable.

## References

[1] Komarek A.M., Dunston S., Enahoro D., Godfray H.C.J., Herrero M., Mason-D’Croz D., Rich K.M., Scarborough P., Springmann M., Sulser T.B. (2021). Income, consumer preferences, and the future of livestock-derived food demand. Glob. Environ. Change.

[2] Hassan M.M., El Zowalaty M.E., Lundkvist Å., Järhult J.D., Khan Nayem M.R., Tanzin A.Z., Badsha M.R., Khan S.A., Ashour H.M. (2021). Residual antimicrobial agents in food originating from animals. Trends Food Sci. Technol..

[3] Schrijver R., Stijntjes M., Rodríguez-Baño J., Tacconelli E., Babu Rajendran N., Voss A. (2018). Review of antimicrobial resistance surveillance programmes in livestock and meat in EU with focus on humans. Clin. Microbiol. Infect..

[4] Tiseo K., Huber L., Gilbert M., Robinson T.P., Van Boeckel T.P. (2020). Global Trends in Antimicrobial Use in Food Animals from 2017 to 2030. Antibiotics.

[5] Cuevas-González P.F., Peredo-Lovillo A., Castro-López C., Vallejo-Cordoba B., González-Córdova A.F., García H.S., Hernández-Mendoza A. (2021). Food-grade lactic acid bacteria and probiotics as a potential protective tool against erythrotoxic dietary xenobiotics. Trends Food Sci. Technol..

[6] Barba-Vidal E., Martín-Orúe S.M., Castillejos L. (2019). Practical aspects of the use of probiotics in pig production: A review. Livest. Sci..

[7] Gibson M.K., Crofts T.S., Dantas G. (2015). Antibiotics and the developing infant gut microbiota and resistome. Curr. Opin. Microbiol..

[8] Tavoukjian V. (2019). Faecalmicrobiota transplantation for the decolonization of antibiotic-resistant bacteria in the gut: A systematic review and meta-analysis. J. Hosp. Infect..

[9] Andremont A., Cervesi J., Bandinelli P.-A., Vitry F., de Gunzburg J. (2021). Spare and repair the gut microbiota from antibiotic-induced dysbiosis: State-of-the-art. Drug Discov. Today.

[10] Tomosada Y., Villena J., Murata K., Chiba E., Shimazu T., Aso H., Iwabuchi N., Xiao J.Z., Saito T., Kitazawa H. (2013). Immunoregulatory effect of bifidobacteria strains in porcine intestinal epithelial cells through modulation of ubiquitin-editing enzyme A20 expression. PLoS ONE.

[11] Suda Y., Villena J., Takahashi Y., Hosoya S., Tomosada Y., Tsukida K., Shimazu T., Aso H., Tohno M., Ishida M. (2014). Immunobiotic Lactobacillus jensenii as immune-health promoting factor to improve growth performance and productivity in post-weaning pigs. BMC Immunol..

[12] Fujie H., Villena J., Tohno M., Morie K., Shimazu T., Aso H., Suda Y., Shimosato T., Iwabuchi N., Xiao J.Z. (2011). Toll-like receptor-2-activating bifidobacteria strains differentially regulate inflammatory cytokines in the porcine intestinal epithelial cell culture system: Finding new anti-inflammatory immunobiotics. FEMS Immunol. Med. Microbiol..

[13] Villena J., Salva S., Núñez M., Corzo J., Tolaba R., Faedda J., Font G., Alvarez S. (2012). Probiotics for Everyone! The Novel Immunobiotic Lactobacillus rhamnosus CRL1505 and the Beginning of Social Probiotic Programs in Argentina. Int. J. Biotechnol. Wellness Ind..

[14] Kumagae N., Villena J., Tomosada Y., Kobayashi H., Kanmani P., Aso H., Sasaki T., Yoshida M., Tanabe H., Shibata I. (2014). Evaluation of the Immunoregulatory Capacities of Feed Microbial Materials in Porcine Intestinal Immune and Epithelial Cells. Open J. Vet. Med..

[15] Kobayashi H., Kanmani P., Ishizuka T., Miyazaki A., Soma J., Albarracin L., Suda Y., Nochi T., Aso H., Iwabuchi N. (2017). Development of an in vitro immunobiotic evaluation system against rotavirus infection in bovine intestinal epitheliocytes. Benef. Microbes.

[16] Dowarah R., Verma A.K., Agarwal N., Patel B.H.M., Singh P. (2017). Effect of swine based probiotic on performance, diarrhoea scores, intestinal microbiota and gut health of grower-finisher crossbred pigs. Livest. Sci..

[17] Kwak M.-J., Tan P.L., Oh J.K., Chae K.S., Kim J., Kim S.H., Eun J.-S., Chee S.W., Kang D.-K., Kim S.H. (2021). The effects of multispecies probiotic formulations on growth performance, hepatic metabolism, intestinal integrity and fecal microbiota in growing-finishing pigs. Anim. Feed Sci. Technol..

[18] Meng Q.W., Yan L., Ao X., Zhou T.X., Wang J.P., Lee J.H., Kim I.H. (2010). Influence of probiotics in different energy and nutrient density diets on growth performance, nutrient digestibility, meat quality, and blood characteristics in growing-finishing pigs. J. Anim. Sci..

[19] Veizaj-Delia E., Piu T., Lekaj P., Tafaj M. (2010). Using combined probiotic to improve growth performance of weaned piglets on extensive farm conditions. Livest. Sci..

[20] Giang H., Viet T., Ogle B., Lindberg J. (2011). Effects of Supplementation of Probiotics on the Performance, Nutrient Digestibility and FaecalMicroflora in Growing-finishing Pigs. Asian-Australas. J. Anim. Sci..

[21] Suo C., Yin Y., Wang X., Lou X., Song D., Wang X., Gu Q. (2012). Effects of lactobacillus plantarum ZJ316 on pig growth and pork quality. BMC Vet. Res..

[22] Herfel T.M., Jacobi S.K., Lin X., Jouni Z.E., Chichlowski M., Stahl C.H., Odle J. (2013). Dietary supplementation of Bifidobacterium longum strain AH1206 increases its cecal abundance and elevates intestinal interleukin-10 expression in the neonatal piglet. Food Chem. Toxicol. Int. J. Publ. Br. Ind. Biol. Res. Assoc..

[23] Liu H., Zhang J., Zhang S., Yang F., Thacker P.A., Zhang G., Qiao S., Ma X. (2014). Oral administration of Lactobacillus fermentum I5007 favors intestinal development and alters the intestinal microbiota in formula-fed piglets. J. Agric. Food Chem..

[24] Sonia T., Ji H., Hong-Seok M., Chul-Ju Y. (2014). Evaluation of Lactobacillus and Bacillus-based probiotics as alternatives to antibiotics in enteric microbial challenged weaned piglets. Afr. J. Microbiol. Res..

[25] Kantas D., Papatsiros V.G., Tassis P.D., Giavasis I., Bouki P., Tzika E.D. (2015). A feed additive containing Bacillus toyonensis (Toyocerin^®^) protects against enteric pathogens in postweaning piglets. J. Appl. Microbiol..

[26] Lan R.X., Lee S.I., Kim I.H. (2016). Effects of multistrain probiotics on growth performance, nutrient digestibility, blood profiles, faecal microbial shedding, faecal score and noxious gas emission in weaning pigs. J. Anim. Physiol. Anim. Nutr..

[27] Barba-Vidal E., Castillejos L., López-Colom P., RiveroUrgell M., Moreno Muñoz J.A., Martín-Orúe S.M. (2017). Evaluation of the Probiotic Strain *Bifidobacterium longum* subsp. *Infantis* CECT 7210 Capacities to Improve Health Status and Fight Digestive Pathogens in a Piglet Model. Front. Microbiol..

[28] Ngo T.T., Bang N.N., Dart P., Callaghan M., Klieve A., Hayes B., McNeill D. (2021). Feed Preference Response of Weaner Bull Calves to Bacillus amyloliquefaciens H57 Probiotic and Associated Volatile Organic Compounds in High Concentrate Feed Pellets. Animals.

[29] Masumizu Y., Zhou B., Kober A.K.M.H., Islam M.A., Iida H., Ikeda-Ohtsubo W., Suda Y., Albarracin L., Nochi T., Aso H. (2019). Isolation and Immunocharacterization of Lactobacillus salivarius from the Intestine of Wakame-Fed Pigs to Develop Novel “Immunosynbiotics”. Microorganisms.

[30] Tan B., Lim T., Boontiam W. (2020). Effect of dietary supplementation with essential oils and a Bacillus probiotic on growth performance, diarrhoea and blood metabolites in weaned pigs. Anim. Prod. Sci..

[31] Islam M.A., Hashiguchi K., Kober A.K.M.H., Morie K., Zhou B., Tomokiyo M., Shimazu T., Aso H., Villena J., Suda Y. (2021). Effect of Dietary Supplementation of ImmunobioticLactiplantibacillusplantarum N14 Fermented Rakkyo (*Allium chinense*) Pickled Juice on the Immunocompetence and Production Performance of Pigs. Animals.

[32] Suda Y., Sasaki N., Kagawa K., Elean M., Zhou B., Tomokiyo M., Islam M.A., Rajoka M.S.R., Kober A.K.M.H., Shimazu T. (2021). Immunobiotic Feed Developed with Lactobacillus delbrueckii subsp. delbrueckii TUA4408L and the Soymilk By-Product Okara Improves Health and Growth Performance in Pigs. Microorganisms.

[33] Tian Z., Cui Y., Lu H., Wang G., Ma X. (2021). Effect of long-term dietary probiotic Lactobacillus reuteri 1 or antibiotics on meat quality, muscular amino acids and fatty acids in pigs. Meat Sci..

[34] Wang H., Kim I.-H. (2021). Evaluation of Dietary Probiotic (*Lactobacillus plantarum* BG0001) Supplementation on the Growth Performance, Nutrient Digestibility, Blood Profile, Fecal Gas Emission, and Fecal Microbiota in Weaning Pigs. Animals.

[35] Frizzo L.S., Zbrun M.V., Soto L.P., Signorini M.L. (2011). Effects of probiotics on growth performance in young calves: A meta-analysis of randomized controlled trials. Anim. Feed Sci. Technol..

[36] Jatkauskas J., Vrotniakiene V. (2014). Effects of encapsulated probiotic Enterococcus faecium strain on diarrhoea patterns and performance of early weaned calves. Vet. Zootech..

[37] Roos T.B., de Moraes C.M., Sturbelle R.T., Dummer L.A., Fischer G., Leite F.P.L. (2018). Probiotics Bacillus toyonensis and Saccharomyces boulardii improve the vaccine immune response to Bovine herpesvirus type 5 in sheep. Res. Vet. Sci..

[38] Tripathi M.K., Karim S.A. (2010). Effect of individual and mixed live yeast culture feeding on growth performance, nutrient utilization and microbial crude protein synthesis in lambs. Anim. Feed Sci. Technol..

[39] Bayatkouhsar J., Tahmasebi A.M., Naserian A.A., Mokarram R.R., Valizadeh R. (2013). Effects of supplementation of lactic acid bacteria on growth performance, blood metabolites and fecal coliform and lactobacilli of young dairy calves. Anim. Feed Sci. Technol..

[40] Soren N.M., Tripathi M.K., Bhatt R.S., Karim S.A. (2013). Effect of yeast supplementation on the growth performance of Malpura lambs. Trop. Anim. Health Prod..

[41] Agazzi A., Tirloni E., Stella S., Maroccolo S., Ripamonti B., Bersani C., Caputo J., Dell’Orto V., Rota N., Savoini G. (2014). Effects of species-specific probiotic addition to milk replacer on calf health and performance during the first month of life. Ann. Anim. Sci..

[42] Foditsch C., Pereira R.V., Ganda E.K., Gomez M.S., Marques E.C., Santin T., Bicalho R.C. (2015). Oral Administration of Faecalibacteriumprausnitzii Decreased the Incidence of Severe Diarrhea and Related Mortality Rate and Increased Weight Gain in Preweaned Dairy Heifers. PLoS ONE.

[43] Zhang R., Zhou M., Tu Y., Zhang N.F., Deng K.D., Ma T., Diao Q.Y. (2016). Effect of oral administration of probiotics on growth performance, apparent nutrient digestibility and stress-related indicators in Holstein calves. J. Anim. Physiol. Anim. Nutr..

[44] Saleem A., Zanouny A., Singer A. (2016). Growth Performance, Nutrients Digestibility, and Blood Metabolites of Lambs Fed Diets Supplemented with Probiotics during Pre- and Post-Weaning Period. Asian-Australas. J. Anim. Sci..

[45] Adjei-Fremah S., Ekwemalor K., Asiamah E.K., Ismail H., Ibrahim S., Worku M. (2018). Effect of probiotic supplementation on growth and global gene expression in dairy cows. J. Appl. Anim. Res..

[46] Sharma A.N., Kumar S., Tyagi A.K. (2018). Effects of mannan-oligosaccharides and Lactobacillus acidophilus supplementation on growth performance, nutrient utilization and faecal characteristics in Murrah buffalo calves. J. Anim. Physiol. Anim. Nutr..

[47] Maldonado N.C., Chiaraviglio J., Bru E., De Chazal L., Santos V., Nader-Macías M.E.F. (2018). Effect of Milk Fermented with Lactic Acid Bacteria on Diarrheal Incidence, Growth Performance and Microbiological and Blood Profiles of Newborn Dairy Calves. Probiotics Antimicrob. Proteins.

[48] Hassan A., Gado H., Anele U.Y., Berasain M.A.M., Salem A.Z.M. (2020). Influence of dietary probiotic inclusion on growth performance, nutrient utilization, ruminal fermentation activities and methane production in growing lambs. Anim. Biotechnol..

[49] Zábranský L. (2021). Effect of prebiotic and probiotic supplements to increase live weight of calves in the diet. Acta Fytotech. Zootech..

[50] Liu F., Li G., Wen K., Bui T., Cao D., Zhang Y., Yuan L. (2010). Porcine small intestinal epithelial cell line (IPEC-J2) of rotavirus infection as a new model for the study of innate immune responses to rotaviruses and probiotics. Viral Immunol..

[51] Kanmani P., Kim H. (2019). Immunobiotic strains modulate toll-like receptor 3 agonist induced innate antiviral immune response in human intestinal epithelial cells by modulating IFN regulatory factor 3 and NF-κB signaling. Front. Immunol..

[52] Takanashi N., Tomosada Y., Villena J., Murata K., Takahashi T., Chiba E., Tohno M., Shimazu T., Aso H., Suda Y. (2013). Advanced application of bovine intestinal epithelial cell line for evaluating regulatory effect of lactobacilli against heat-killed enterotoxigenic Escherichia coli-mediated inflammation. BMC Microbiol..

[53] Abedi D., Feizizadeh S., Akbari V., Jafarian-Dehkordi A. (2013). In vitro anti-bacterial and anti-adherence effects of Lactobacillus delbrueckii subsp bulgaricus on Escherichia coli. Res. Pharm. Sci..

[54] Shimazu T., Villena J., Tohno M., Fujie H., Hosoya S., Shimosato T., Aso H., Suda Y., Kawai Y., Saito T. (2012). Immunobiotic Lactobacillus jensenii elicits anti-inflammatory activity in porcine intestinal epithelial cells by modulating negative regulators of the Toll-like receptor signaling pathway. Infect. Immun..

[55] Lim H.J., Shin H.S. (2021). Antimicrobial and immunomodulatory effects of bifidobacterium strains: A review. J. Microbiol. Biotechnol..

[56] Villena J., Chiba E., Vizoso-Pinto M.G., Tomosada Y., Takahashi T., Ishizuka T., Aso H., Salva S., Alvarez S., Kitazawa H. (2014). Immunobiotic Lactobacillus rhamnosus strains differentially modulate antiviral immune response in porcine intestinal epithelial and antigen presenting cells. BMC Microbiol..

[57] Kang J.Y., Lee D.K., Ha N.J., Shin H.S. (2015). Antiviral effects of Lactobacillus ruminis SPM0211 and Bifidobacterium longum SPM1205 and SPM1206 on rotavirus-infected Caco-2 cells and a neonatal mouse model. J. Microbiol..

[58] Laiño J., Villena J., Kanmani P., Kitazawa H. (2016). Immunoregulatory Effects Triggered by Lactic Acid Bacteria Exopolysaccharides: New Insights into Molecular Interactions with Host Cells. Microorganisms.

[59] Kanmani P., Albarracin L., Kobayashi H., Iida H., Komatsu R., Humayun Kober A.K.M., Ikeda-Ohtsubo W., Suda Y., Aso H., Makino S. (2018). Exopolysaccharides from Lactobacillus delbrueckii OLL1073R-1 modulate innate antiviral immune response in porcine intestinal epithelial cells. Mol. Immunol..

[60] Iida H., Tohno M., Islam M.A., Sato N., Kobayashi H., Albarracin L., Kober A.H., Ikeda-Ohtsubo W., Suda Y., Aso H. (2019). Paraimmunobiotic Bifidobacteria Modulate the Expression Patterns of Peptidoglycan Recognition Proteins in Porcine Intestinal Epitheliocytes and Antigen Presenting Cells. Cells.

[61] Mizuno H., Arce L., Tomotsune K., Albarracin L., Funabashi R., Vera D., Islam M.A., Vizoso-Pinto M.G., Takahashi H., Sasaki Y. (2020). Lipoteichoic Acid Is Involved in the Ability of the Immunobiotic Strain Lactobacillus plantarum CRL1506 to Modulate the Intestinal Antiviral Innate Immunity Triggered by TLR3 Activation. Front. Immunol.

[62] Śliżewska K., Chlebicz-Wójcik A., Nowak A. (2021). Probiotic Properties of New Lactobacillus Strains Intended to Be Used as Feed Additives for Monogastric Animals. Probiotics Antimicrob. Proteins.

[63] Van Boeckel T.P., Brower C., Gilbert M., Grenfell B.T., Levin S.A., Robinson T.P., Teillant A., Laxminarayan R. (2015). Global trends in antimicrobial use in food animals. Proc. Natl. Acad. Sci. USA.

[64] De Rycker M., Baragaña B., Duce S.L., Gilbert I.H. (2018). Challenges and recent progress in drug discovery for tropical diseases. Nature.

[65] Álvarez-Ordóñez A., Carvajal A., Arguello H., Martínez-Lobo F.J., Naharro G., Rubio P. (2013). Antibacterial activity and mode of action of a commercial citrus fruit extract. J. Appl. Microbiol..

[66] Wang H., Ha B., Kim I.H. (2021). Effects of probiotics complex supplementation in low nutrient density diet on growth performance, nutrient digestibility, faecal microbial, and faecal noxious gas emission in growing pigs. Ital. J. Anim. Sci..

[67] Méndez-Palacios N., Méndez-Mendoza M., Vázquez-Flores F., Castro-Colombres J.G., Ramírez-Bribiesca J.E. (2018). Productive and economic parameters of pigs supplemented from weaning to finishing with prebiotic and probiotic feed additives. Anim. Sci. J..

[68] Zommiti M., Chikindas M.L., Ferchichi M. (2020). Probiotics—Live Biotherapeutics: A Story of Success, Limitations, and Future Prospects—Not Only for Humans. Probiotics Antimicrob. Proteins.

[69] Tang Q., Yi H., Hong W., Wu Q., Yang X., Hu S., Xiong Y., Wang L., Jiang Z. (2021). Comparative Effects of L. plantarum CGMCC 1258 and L. reuteri LR1 on Growth Performance, Antioxidant Function, and Intestinal Immunity in Weaned Pigs. Front. Vet. Sci..

[70] Raabis S., Li W., Cersosimo L. (2019). Effects and immune responses of probiotic treatment in ruminants. Vet. Immunol. Immunopathol..

[71] Flint H.J. (1997). The rumen microbial ecosystem—some recent developments. Trends Microbiol..

[72] Andersen T.O., Kunath B.J., Hagen L.H., Arntzen M.Ø., Pope P.B. (2021). Rumen metaproteomics: Closer to linking rumen microbial function to animal productivity traits. Methods.

[73] Cagle C.M., Fonseca M.A., Callaway T.R., Runyan C.A., Cravey M.D., Tedeschi L.O. (2020). Evaluation of the effects of live yeast on rumen parameters and in situ digestibility of dry matter and neutral detergent fiber in beef cattle fed growing and finishing diets. Appl. Anim. Sci..

[74] Sousa D.O., Oliveira C.A., Velasquez A.V., Souza J.M., Chevaux E., Mari L.J., Silva L.F.P. (2018). Live yeast supplementation improves rumen fibre degradation in cattle grazing tropical pastures throughout the year. Anim. Feed Sci. Technol..

[75] Rossow H., Riordan T., Riordan A. (2017). Effects of addition of a live yeast product on dairy cattle performance. J. Appl. Anim. Res..

[76] Maamouri O., Ben Salem M. (2021). Effect of yeast culture feed supply on growth, ruminal pH, and digestibility of fattening calves. Food Sci. Nutr..

[77] Frizzo L., Bertozzi E., Soto L.P., Zbrun M., Sequeira G., Santina R., Armesto R., Rosmini M. (2008). The Effect of Supplementation with Three Lactic Acid Bacteria from Bovine Origin on Growth Performance and Health Status of Young Calves. J. Anim. Vet. Adv..

[78] Arowolo M.A., He J. (2018). Use of probiotics and botanical extracts to improve ruminant production in the tropics: A review. Anim. Nutr..

[79] Uyeno Y., Shigemori S., Shimosato T. (2015). Effect of probiotics/prebiotics on cattle health and productivity. Microbes Environ..

[80] Colombo E.A., Cooke R.F., Brandão A.P., Wiegand J.B., Schubach K.M., Sowers C.A., Duff G.C., Block E., Gouvêa V.N. (2021). Performance, health, and physiological responses of newly received feedlot cattle supplemented with pre- and probiotic ingredients. Animal.

[81] Batista L.H.C., Cidrini I.A., Prados L.F., Cruz A.A.C., Torrecilhas J.A., Siqueira G.R., Resende F.D. (2022). A meta-analysis of yeast products for beef cattle under stress conditions: Performance, health and physiological parameters. Anim. Feed Sci. Technol..

[82] Wang H., Yu Z., Gao Z., Li Q., Qiu X., Wu F., Guan T., Cao B., Su H. (2021). Effects of compound probiotics on growth performance, rumen fermentation, blood parameters, and health status of neonatal Holstein calves. J. Dairy Sci..

[83] Górka P., Budzińska K., Budziński W., Jankowiak T., Kehoe S., Kański J. (2021). Effect of probiotic and nucleotide supplementation in milk replacer on growth performance and fecal bacteria in calves. Livest. Sci..

[84] Alawneh J.I., Barreto M.O., Moore R.J., Soust M., Al-harbi H., James A.S., Krishnan D., Olchowy T.W.J. (2020). Systematic review of an intervention: The use of probiotics to improve health and productivity of calves. Prev. Vet. Med..

[85] Izadi b., MohebbiFani M., Hosseinzadeh S., Shekarforoush S.S., Rasooli A., Nazifi S. (2020). Effect of Bacillus coagulans probiotic on milk production and important economic and health indicators of raw milk of Holstein cows. Iran. Vet. J..

[86] Endo K., Mine Y., Shuto T., Taji T., Murayama T., Nikawa H. (2020). Comprehensive analysis of transcriptional profiles in oral epithelial-like cells stimulated with oral probiotic *Lactobacillus* spp.. Arch. Oral Biol..

[87] Vale G.C., Mayer M.P.A. (2021). Effect of probiotic Lactobacillus rhamnosus by-products on gingival epithelial cells challenged with Porphyromonasgingivalis. Arch. Oral Biol..

[88] Moue M., Tohno M., Shimazu T., Kido T., Aso H., Saito T., Kitazawa H. (2008). Toll-like receptor 4 and cytokine expression involved in functional immune response in an originally established porcine intestinal epitheliocyte cell line. Biochim. Biophys. Acta.

[89] Miyazawa K., Hondo T., Kanaya T., Tanaka S., Takakura I., Itani W., Rose M.T., Kitazawa H., Yamaguchi T., Aso H. (2010). Characterization of newly established bovine intestinal epithelial cell line. Histochem. Cell Biol..

[90] Hosoya S., Villena J., Shimazu T., Tohno M., Fujie H., Chiba E., Shimosato T., Aso H., Suda Y., Kawai Y. (2011). Immunobiotic lactic acid bacteria beneficially regulate immune response triggered by poly(I:C) in porcine intestinal epithelial cells. Vet. Res..

[91] Wachi S., Kanmani P., Tomosada Y., Kobayashi H., Yuri T., Egusa S., Shimazu T., Suda Y., Aso H., Sugawara M. (2014). Lactobacillus delbrueckii TUA4408L and its extracellular polysaccharides attenuate enterotoxigenic Escherichia coli-induced inflammatory response in porcine intestinal epitheliocytes via Toll-like receptor-2 and 4. Mol. Nutr. Food Res..

[92] Albarracin L., Kobayashi H., Iida H., Sato N., Nochi T., Aso H., Salva S., Alvarez S., Kitazawa H., Villena J. (2017). Transcriptomic Analysis of the Innate Antiviral Immune Response in Porcine Intestinal Epithelial Cells: Influence of Immunobiotic Lactobacilli. Front. Immunol..

[93] Sun Y., Duarte M.E., Kim S.W. (2021). Dietary inclusion of multispecies probiotics to reduce the severity of post-weaning diarrhea caused by *Escherichia coli* F18(+) in pigs. Anim. Nutr./Zhongguoxu Mu Shouyixuehui.

[94] Sobrino O.J., Alba C., Arroyo R., Pérez I., Sariego L., Delgado S., Fernández L., de María J., Fumanal P., Fumanal A. (2021). Replacement of Metaphylactic Antimicrobial Therapy by Oral Administration of *Ligilactobacillus salivarius* MP100 in a Pig Farm. Front. Vet. Sci..

[95] Dou S., Gadonna-Widehem P., Rome V., Hamoudi D., Rhazi L., Lakhal L., Larcher T., Bahi-Jaber N., Pinon-Quintana A., Guyonvarch A. (2017). Characterisation of Early-Life Fecal Microbiota in Susceptible and Healthy Pigs to Post-Weaning Diarrhoea. PLoS ONE.

[96] Yang Q., Huang X., Zhao S., Sun W., Yan Z., Wang P., Li S., Huang W., Zhang S., Liu L. (2017). Structure and Function of the Fecal Microbiota in Diarrheic Neonatal Piglets. Front. Microbiol..

[97] Ratajczak W., Rył A., Mizerski A., Walczakiewicz K., Sipak O., Laszczyńska M. (2019). Immunomodulatory potential of gut microbiome-derived short-chain fatty acids (SCFAs). Acta Biochim. Pol..

[98] Sun J., Du L., Li X., Zhong H., Ding Y., Liu Z., Ge L. (2019). Identification of the core bacteria in rectums of diarrheic and non-diarrheic piglets. Sci. Rep..

[99] Liu Y., Zheng Z., Yu L., Wu S., Sun L., Wu S., Xu Q., Cai S., Qin N., Bao W. (2019). Examination of the temporal and spatial dynamics of the gut microbiome in newborn piglets reveals distinct microbial communities in six intestinal segments. Sci. Rep..

[100] Gruninger R.J., Puniya A.K., Callaghan T.M., Edwards J.E., Youssef N., Dagar S.S., Fliegerova K., Griffith G.W., Forster R., Tsang A. (2014). Anaerobic fungi (phylum Neocallimastigomycota): Advances in understanding their taxonomy, life cycle, ecology, role and biotechnological potential. FEMS Microbiol. Ecol..

[101] Hess M., Paul S.S., Puniya A.K., van der Giezen M., Shaw C., Edwards J.E., Fliegerová K. (2020). Anaerobic Fungi: Past, Present, and Future. Front. Microbiol..

[102] Hagen L.H., Brooke C.G., Shaw C.A., Norbeck A.D., Piao H., Arntzen M.Ø., Olson H.M., Copeland A., Isern N., Shukla A. (2021). Proteome specialization of anaerobic fungi during ruminal degradation of recalcitrant plant fiber. ISME J..

[103] Ruiz Sella S.R.B., Bueno T., de Oliveira A.A.B., Karp S.G., Soccol C.R. (2021). Bacillus subtilis natto as a potential probiotic in animal nutrition. Crit. Rev. Biotechnol..

[104] Cai L., Yu J., Hartanto R., Qi D. (2021). Dietary Supplementation with Saccharomyces cerevisiae, Clostridium butyricum and Their Combination Ameliorate Rumen Fermentation and Growth Performance of Heat-Stressed Goats. Animals.

[105] Angulo M., Reyes-Becerril M., Medina-Córdova N., Tovar-Ramírez D., Angulo C. (2020). Probiotic and nutritional effects of Debaryomyces hansenii on animals. Appl. Microbiol. Biotechnol..

[106] Riaz Rajoka M.S., Thirumdas R., Mehwish H.M., Umair M., Khurshid M., Hayat H.F., Phimolsiripol Y., Pallarés N., Martí-Quijal F.J., Barba F.J. (2021). Role of Food Antioxidants in Modulating Gut Microbial Communities: Novel Understandings in Intestinal Oxidative Stress Damage and Their Impact on Host Health. Antioxidants.

[107] Saleem M., Malik S., Mehwish H.M., Ali M.W., Hussain N., Khurshid M., Rajoka M.S.R., Chen Y. (2021). Isolation and functional characterization of exopolysaccharide produced by Lactobacillus plantarum S123 isolated from traditional Chinese cheese. Arch. Microbiol..

[108] Riaz Rajoka M.S., Mehwish H.M., Xiong Y., Song X., Hussain N., Zhu Q., He Z. (2021). Gut microbiota targeted nanomedicine for cancer therapy: Challenges and future considerations. Trends Food Sci. Technol..

[109] Riaz Rajoka M.S., Wu Y., Mehwish H.M., Bansal M., Zhao L. (2020). Lactobacillus exopolysaccharides: New perspectives on engineering strategies, physiochemical functions, and immunomodulatory effects on host health. Trends Food Sci. Technol..

[110] Riaz Rajoka M.S., Mehwish H.M., Zhang H., Ashraf M., Fang H., Zeng X., Wu Y., Khurshid M., Zhao L., He Z. (2020). Antibacterial and antioxidant activity of exopolysaccharide mediated silver nanoparticle synthesized by Lactobacillus brevis isolated from Chinese koumiss. Colloids Surf. B Biointerfaces.

[111] Riaz Rajoka M.S., Mehwish H.M., Fang H., Padhiar A.A., Zeng X., Khurshid M., He Z., Zhao L. (2019). Characterization and anti-tumor activity of exopolysaccharide produced by Lactobacillus kefiri isolated from Chinese kefir grains. J. Funct. Foods.

[112] Riaz Rajoka M.S., Zhao H., Mehwish H.M., Li N., Lu Y., Lian Z., Shao D., Jin M., Li Q., Zhao L. (2019). Anti-tumor potential of cell free culture supernatant of Lactobacillus rhamnosus strains isolated from human breast milk. Food Res. Int..

[113] Riaz Rajoka M.S., Zhao H., Lu Y., Lian Z., Li N., Hussain N., Shao D., Jin M., Li Q., Shi J. (2018). Anticancer potential against cervix cancer (HeLa) cell line of probiotic Lactobacillus casei and Lactobacillus paracasei strains isolated from human breast milk. Food Funct..

[114] Rajoka M.S.R., Mehwish H.M., Hayat H.F., Hussain N., Sarwar S., Aslam H., Nadeem A., Shi J. (2019). Characterization, the Antioxidant and Antimicrobial Activity of Exopolysaccharide Isolated from Poultry Origin Lactobacilli. Probiotics Antimicrob. Proteins.

[115] Mountzouris K.C., Balaskas C., Xanthakos I., Tzivinikou A., Fegeros K. (2009). Effects of a multi-species probiotic on biomarkers of competitive exclusion efficacy in broilers challenged with Salmonella enteritidis. Br. Poult. Sci..

[116] Zhao P.Y., Kim I.H. (2015). Effect of direct-fed microbial on growth performance, nutrient digestibility, fecal noxious gas emission, fecal microbial flora and diarrhea score in weanling pigs. Anim. Feed Sci. Technol..

[117] Walker W.A. (2000). Role of nutrients and bacterial colonization in the development of intestinal host defense. J. Pediatr. Gastroenterol. Nutr..

[118] Dowarah R., Verma A.K., Agarwal N. (2017). The use of Lactobacillus as an alternative of antibiotic growth promoters in pigs: A review. Anim. Nutr./Zhongguoxu Mu Shouyixuehui.

[119] Johnson-Henry K.C., Hagen K.E., Gordonpour M., Tompkins T.A., Sherman P.M. (2007). Surface-layer protein extracts from Lactobacillus helveticus inhibit enterohaemorrhagic Escherichia coli O157:H7 adhesion to epithelial cells. Cell. Microbiol..

[120] Lessard M., Dupuis M., Gagnon N., Nadeau E., Matte J., Goulet J., Fairbrother J. (2008). Administration of Pediococcus Acidilactici or Saccharomyces CerevisiaeBoulardii modulates development of porcine mucosal immunity and reduces intestinal bacterial translocation after Escherichia Coli challenge. J. Anim. Sci..

[121] Sato N., Garcia-Castillo V., Yuzawa M., Islam M.A., Albarracin L., Tomokiyo M., Ikeda-Ohtsubo W., Garcia-Cancino A., Takahashi H., Villena J. (2020). Immunobiotic Lactobacillus jensenii TL2937 Alleviates Dextran Sodium Sulfate-Induced Colitis by Differentially Modulating the Transcriptomic Response of Intestinal Epithelial Cells. Front. Immunol..

[122] Gangadharan D., Sivaramakrishnan S., Nampoothiri K.M., Sukumaran R.K., Pandey A. (2008). Response surface methodology for the optimization of alpha amylase production by Bacillus amyloliquefaciens. Bioresour. Technol..

[123] Lee Y.J., Kim B.K., Lee B.H., Jo K.I., Lee N.K., Chung C.H., Lee Y.C., Lee J.W. (2008). Purification and characterization of cellulase produced by Bacillus amyoliquefaciens DL-3 utilizing rice hull. Bioresour. Technol..

[124] Fayol-Messaoudi D., Berger C.N., Coconnier-Polter M.H., Liévin-Le Moal V., Servin A.L. (2005). pH-, Lactic acid-, and non-lactic acid-dependent activities of probiotic Lactobacilli against Salmonella enterica Serovar Typhimurium. Appl. Environ. Microbiol..

[125] Daşkıran M., Önol A.G., Cengiz Ö., Ünsal H., Türkyılmaz S., Tatlı O., Sevim Ö. (2012). Influence of dietary probiotic inclusion on growth performance, blood parameters, and intestinal microflora of male broiler chickens exposed to posthatch holding time. J. Appl. Poult. Res..

[126] Higgins D.A., Pomianek M.E., Kraml C.M., Taylor R.K., Semmelhack M.F., Bassler B.L. (2007). The major Vibrio cholerae autoinducer and its role in virulence factor production. Nature.

[127] Pringsulaka O., Rueangyotchanthana K., Suwannasai N., Watanapokasin R., Amnueysit P., Sunthornthummas S., Sukkhum S., Sarawaneeyaruk S., Rangsiruji A. (2015). In vitro screening of lactic acid bacteria for multi-strain probiotics. Livest. Sci..

[128] Oh B.-T., Jeong S.-Y., Velmurugan P., Park J.-H., Jeong D.-Y. (2017). Probiotic-mediated blueberry (*Vaccinium corymbosum* L.) fruit fermentation to yield functionalized products for augmented antibacterial and antioxidant activity. J. Biosci. Bioeng..

[129] Jana U.K., Suryawanshi R.K., Prajapati B.P., Kango N. (2021). Prebiotic mannooligosaccharides: Synthesis, characterization and bioactive properties. Food Chem..

[130] García C., Rendueles M., Díaz M. (2019). Liquid-phase food fermentations with microbial consortia involving lactic acid bacteria: A review. Food Res. Int..

